# Impact of oestrus synchronization devices on ewes vaginal microbiota and artificial insemination outcome

**DOI:** 10.3389/fmicb.2023.1063807

**Published:** 2023-03-23

**Authors:** Edgar L. Reinoso-Peláez, María Saura, Óscar González-Recio, Carmen González, Almudena Fernández, Ramón Peiro-Pastor, Adrián López-García, Alejandro Saborío-Montero, Jorge H. Calvo, Manuel Ramón, Malena Serrano

**Affiliations:** ^1^Departamento de Mejora Genética Animal, Instituto Nacional de Investigación y Tecnología Agraria y Alimentaria (INIA-CSIC), Madrid, Spain; ^2^Departamento de Producción Agraria, Escuela Técnica Superior de Ingeniería Agronómica, Alimentaria y de Biosistemas, Universidad Politécnica de Madrid, Madrid, Spain; ^3^Departamento de Ciencia Animal, Centro de Investigación y Tecnología Agroalimentaria de Aragón (CITA-ARAID-IA2), Zaragoza, Spain; ^4^Departamento de Investigación en Reproducción y Mejora Genética Animal, Centro Regional de Selección y Reproducción Animal de Castilla La Mancha (CERSYRA-IRIAF), Valdepeñas, Spain

**Keywords:** artificial insemination, fertility, nanopore, ovine, reproduction, vaginal microbiota, metagenome, microbiome

## Abstract

**Introduction:**

The low pregnancy rate by artificial insemination in sheep represents a fundamental challenge for breeding programs. In this species, oestrus synchronization is carried out by manipulating hormonal regimens through the insertion of progestogen intravaginal devices. This reproductive strategy may alter the vaginal microbiota affecting the artificial insemination outcome.

**Methods:**

In this study, we analyzed the vaginal microbiome of 94 vaginal swabs collected from 47 ewes with alternative treatments applied to the progesterone-releasing intravaginal devices (probiotic, maltodextrin, antibiotic and control), in two sample periods (before placing and after removing the devices). To our knowledge, this is the first study using nanopore-based metagenome sequencing for vaginal microbiome characterization in livestock.

**Results:**

Our results revealed a significant lower abundance of the genera *Oenococcus* (Firmicutes) and *Neisseria* (Proteobacteria) in pregnant compared to non-pregnant ewes. We also detected a significant lower abundance of *Campylobacter* in the group of samples treated with the probiotic.

**Discussion:**

Although the use of probiotics represents a promising practice to improve insemination results, the election of the suitable species and concentration requires further investigation. In addition, the use of progestogen in the synchronization devices seemed to increase the alpha-diversity and decrease the abundance of harmful microorganisms belonging to Gammaproteobacteria and Fusobacteriia classes, suggesting a beneficial effect of their use.

## 1. Introduction

Ovine production has a great economic relevance in Spain, which is the second largest producer of sheep products in Europe and the fifth worldwide. Fertility in sheep farming plays a fundamental role in its profitability and is one of the most important challenges in the sector. Artificial insemination (AI) is the key technology in dairy ruminants breeding programs for progeny testing, connecting herds, and disseminating genetic improvement. Thus, systematic use of AI increases the selection response compared to systems where natural mating is the only method for reproduction. In large-scale dairy sheep breeding farms, AI is performed at a fixed time. This requires therefore the oestrus synchronization of ewes, to ensure they come into heat and to synchronize lambing of the inseminated ewe batch. However, the AI fertility rate in small ruminants -particularly in sheep- is low (30%–70%) compared to other livestock species (Cseh et al., 2012; Vilariño et al., 2013; Granleese et al., 2015). This low efficiency is partially due to the particular morphology of the reproductive tract of the ewe, the need to use fresh semen, and the difficulty to determine the exact stage of the ewe ovulatory cycle when insemination is performed (Alvarez et al., 2019). Some strategies have been addressed to improve the AI success rate, most of them focused mainly on the AI technique, nutrition, health, and oestrus synchronization methods. These advances have led to a slight increase in the AI pregnancy rate. However, our understanding about other potential factors that may affect the success of AI in sheep is still low.

In sheep, a typical practice for oestrus synchronization is the alteration of ewes hormonal regime through the use of progesterone-releasing intravaginal devices (PRID) (Hameed et al., 2021). However, the long-term use of intravaginal sponges is often related to vaginitis and purulent discharge (Martinez-Ros et al., 2018), which has promoted the use of antibiotics (locally or systemic) at the time of sponge application (Gatti et al., 2011). Probiotics (*Lactobacillus*) have been used in humans to promote a favorable development of the vaginal microbiota, reducing the increase of pathogenic colonies (Bertuccini et al., 2017; Ratner et al., 2019), whereas their effect in sheep (Quereda et al., 2020) and other livestock species is still unexplored. In particular, previous studies in humans have found that *Lactobacillus rhamnosus* has a beneficial effect on fertility (Reid et al., 1995; Cribby et al., 2008; Kirjavainen et al., 2008; Bertuccini et al., 2017; Ratner et al., 2019), contributing to improve the vaginal environment for local bacteria and to decrease the presence of pathogenic anaerobic microorganisms.

In recent years, the study of the composition and abundance of microbial communities (microbiota) has been facilitated by the advances of high-throughput sequencing technologies. In particular, metagenomics has emerged as the reference technique to analyze the genomes contained in an environmental sample (microbiome). Traditional characterization of the microbiota was based on culture techniques plus phenotypic identification or Sanger sequencing of individual isolates. However, only about 1% of bacteria are readily culturable on common media under standard conditions (Handelsman, 2004). Amplification and (partial or total) sequencing of the 16S ribosomal RNA (rRNA) gene serves as a molecular fingerprint for taxonomic identification (Eckburg et al., 2005) and is currently the most extended technique for microbiome characterization. Notwithstanding, interesting options have been developed that could revolutionize the identification of microorganisms, such as the third-generation massive sequencing. In particular, nanopore sequencing (Oxford Nanopore) works by monitoring changes in electrical current as nucleic acids pass through a protein nanopore, which allows not only taxonomic identification through similarity of sequences but also to identify gene functions and higher-order functional information through KEGGs (Kyoto Encyclopedia of Genes and Genomes) and COG (Clusters of Orthologous Groups) databases, which allow to deepen the studies and interactions of the metagenomic universe in the target populations (Suárez Moya, 2017).

While there is strong evidence of the role of the reproductive tract microbiota on fertility and sexual disease in humans (Hong et al., 2020), its study in livestock species, particularly in sheep, is very limited.

The aim of this study was to elucidate the role of the vaginal microbiota in fertility of Assaf sheep using metagenomic nanopore sequencing. In addition, we evaluated the effect of PRIDs supplemented with different additives on the microbiota composition, and whether potential changes in that composition are related to the pregnancy rate.

## 2. Materials and methods

The current study was carried out under a Project License from the INIA Scientific Ethic Committee. Animal manipulations were performed according to the Spanish Policy for Animal Protection RD 53/2013, which meets the European Union Directive 2010/63/EU about the protection of animals used in experimentation. We hereby confirm that the INIA Scientific Ethic Committee (IACUC) has approved this study.

### 2.1. Animal samples

Fourty-seven Assaf ewes ageing between two and 5 years were selected from the “Pago los Vivales” farm, all of which had lambed one or more times. All ewes were oestrus synchronized with PRIDs containing 20 mg of Flurogestone acetate (Chronogest. MSD Animal Health, Kenilworth, NJ, USA). Before PRIDs placement, a vaginal exudate sample (S1) was taken from each ewe 14 days before AI, with a double sterile swab-tube (BD Culture Swab, Becton Dickinson, Becton Sparks, MD 21152 USA) adding in the tube 3 ml of DNA/RNA Shield (Zymo Research Corporation). Swabs were kept refrigerated until arrival to the laboratory where they were preserved until the extraction at −80°C. We used a speculum to facilitate the sampling, which was disinfected with povidone iodine solution for each single ewe to avoid cross contamination. The ewes were divided in four batches, depending on the treatment added to the PRID: (i) probiotic (*n* = 13), containing 200 mg/PRID of lyophilized *L. rhamnosus* and maltodextrin as excipient (ADM Biopolis, 46,980, Paterna, Valencia, Spain); (ii) maltodextrin (*n* = 10), containing 200 mg/PRID of lyophilized excipient; (iii) antibiotic (*n* = 13), containing 0.6 gr/PRID of Framitecin in power (neomycin sulfate, Framicas. Laboratorios Ovejero, Spain), and (iv) control (*n* = 11), i.e., no additive added to the PRID.

Due to the scarcity of information on microbial communities and probiotics in sheep, *L. rhamnosus* was used here as a starting point in the search for probiotics with a positive synergistic effect to improve the vaginal microbial environment of sheep.

After 14 days, PRIDs were removed and immediately ewes were injected with a dose of 300 to 500 mg of PMSG (pregnant mare’s serum gonadotropin) depending on body weight, to stimulate ovulation. Artificial insemination was conducted 53–55 h after PRID removal. Just before insemination, a second sample (S2) of vaginal exudate was taken from each ewe in the same way as S1. Ewes were inseminated with fresh semen from four rams belonging to OVIGEN insemination center. Rams used for AI aged between 4 and 7 years, and started being semen donors at 10 months of age. Sperm doses were prepared with fresh semen at a concentration of 400 million of spermatozoids/mL using as diluent INRA96® (IMV Technologies, L’Aigle, France), plus 50 mg of streptomycin and 50,000 IU/ml of diluent of penicillin and packed in 0.25 ml straws. Inseminations were performed within 5 h of sperm straw preparation, maintaining semen straws at 15°C. A complete factorial design was performed, to obtain a similar number of ewes inseminated with the four rams within the four treatment groups. Pregnancy was determined by ultrasound 42 days after AI.

### 2.2. DNA extraction and sequencing

Swabs impregnated with vaginal exudate were cut and immersed in individual Eppendorf tubes. DNA from vaginal swabs were extracted with the QIAamp ©DNA Microbiome extraction kit (Qiagen Inc., Valencia, CA, United States) following the protocol instructions. DNA samples were eluted in 15 to 30 μl DEPC water. Genomic DNA concentration and quality ratios 260/280 and 260/230 were measured using a Qubit 4 fluorometer (Thermo Fisher Scientific, DE, United States) and a Nanodrop 2000 spectrophotometer (Thermo Fisher Scientific, DE, United States), respectively.

Microbial DNA sequencing was carried out through nanopore technology using a MinION sequencer (Oxford Nanopore Technologies, ONT). One μg of DNA from each sample was used as initial material for sequencing, following the ligation sequencing kit (SQK-LSK109) protocol. Twelve samples were multiplexed in each run with the 1D Native Barcoding genomic DNA kit (EXP-NBD104 and EXP-NBD114). The barcoded samples (700 ng of DNA in total) were pooled in a 1.5-mL Eppendorf DNA LoBind tube to perform adapter ligation for sequencing using R9.4.1 flow cells. To sequence the 93 microbial DNA samples, eight flow cells were used.

### 2.3. Bioinformatic analysis

Basecalling was performed with the Guppy 4.2.2 software provided by ONT. In order to remove reads from the host, a filtering step was performed by mapping sequences against the *Ovis aries* reference genome from NCBI (Oar_rambouillet_v1.0. GCA_002742125.1) using BBMap software^1^. After removing the host genome, 5.5 Gb corresponding to 1,890,475 reads were retained, with an average read length per sample of 2,930.36 bp and an average number of reads per sample of 20,111. Retained reads were analyzed using the SqueezeMeta 1.3.0 pipeline for long reads (Tamames and Puente-Sánchez, 2019), which performs Diamond Blastx against NCBI-nr, KEGG (Kyoto Enciclopedia of Genes and Genomes) and COG (Clusters of Orthologous Genes) databases. SqueezeMeta implements a lowest common ancestor (LCA) algorithm to find the consensus taxon for each read. This pipeline also aligns each read to a gene reference database and provides the number of copies of each gene present in the sample. Gene functions were annotated using the best hit above a minimum score threshold of 60 (genus), 55 (family), 50 (order), 46 (class), and 42 (phylum), which are the default values of SqueezeMeta software. Hits below these thresholds were considered as unclassified taxa. In addition, taxa not belonging to bacteria, archaeobacteria, virus, fungi and protozoa were manually filtered out from the dataset, those reads belonging to taxa with a prevalence threshold <0.05% at the genus level and < 0.01% at the phylum level were discarded in order to reduce data sparsity. These filter values have been chosen according to the number of taxa available and the compatibility with the zero-imputation.

### 2.4. Microbial composition analysis

The diversity and abundance of the vaginal microbiota of the samples grouped by pregnancy status (pregnant/non-pregnant) or by sampling points (S1 vs. S2) were assessed estimating the alpha and beta diversity. Notice that the data available for the comparison of sampling groups comprises all 94 samples obtained from the 47 ewes, while the comparison of pregnancy groups only includes the 47 samples from sampling point S2 (i.e., those with the PRID treatments).

Four alpha-diversity measures were estimated: (i) observed richness, the total number of species in a sample, (ii) Chao1 index, the number of species weighted by the number of rare species in the sample, (iii) Shannon index, the number of species weighted by their abundance and evenness of distribution, and (iv) inverse Simpson index, a measure of diversity that considers both the number of species present as well as their abundance. Alpha-diversity was computed by using the function estimate_richnes from phyloseq (McMurdie and Holmes, 2013).

ANOVA for each alpha-diversity measure was evaluated to determine the existence of differences between groups, by fitting a regression model for each of the three comparisons using the STAT R package (R Core Team, 2019) as follows:


$$y_{i}=\mu +b_{ij}+e_{ij}$$

being $\alpha_{i}$ the alpha-diversity index for each sample *i*, $b_{ij}$ the pregnancy status (with *j* = 2 levels, pregnant and non-pregnant), the S1-S2 group status (with *j* = 2 levels, before or after PRID) or the treatment (with *j* = 4 levels), and $e_{ij}$ the residual.

Beta-diversity analyses were conducted through (i) Principal Component Analysis (PCA), using the FactoMineR R package (Le et al., 2008) and (ii) Permutational Multivariate Analysis of Variance (PERMANOVA) using the Vegan R package (Oksanen et al., 2020). Calculations were based on the Anderson’s algorithm (Anderson, 2001) though the partition of sums of squares using dissimilarities.

Finally, differential abundance analysis between pregnant and non-pregnant ewes and S1 and S2 time point samples were carried out using the Limma R package (Ritchie et al., 2015) by fitting linear models. Multiple testing correction was performed using a Bayesian method. Differential abundance was defined as those taxa showing a |log_2_ fold change (FC)| > 1 and a false discovery rate (FDR) < 0.05.


$$y_{ijkl}=\mu +b_{ij}+t_{ik}+e_{ijkl}$$

Where *y* is the microbial relative abundance of each sample *i*, $b_{ij}$ the pregnancy status (with *j* = 2 levels, pregnant and non-pregnant), or the S1–S2 group (with *j* = 2 levels, before or after PRID), $t_{ik}$ the treatment (with *k* = 4 levels), and $e_{ij}$ the residual. A mixed model for pregnancy fitting only fixed (treatment) and random (ram) effects was previously run to evaluate the inclusion in the model of the ram effect, which was discarded as it was not significant.

To accommodate the compositional nature of metagenomic data, a centered log ratio (CLR) transformation method was implemented for the estimation of the beta-diversity as well as the differential abundance analysis, using the unweighted option of the CLR function from the easyCODA R package (Greenacre, 2018). Count zero values in the initial raw data were imputed to allow computing logarithms. The imputation was done using a Bayesian multiplicative replacement procedure. This procedure was performed with the geometric Bayesian multiplicative method from the cmultRepl function of the zCompositions R package (Palarea-Albaladejo and Martín-Fernández, 2015).

In order to assess the homogeneity of groups, a PERMANOVA between pregnant and non-pregnant groups within S1 (Supplementary Table S7) was performed. The absence of significant results validated the absence of bias due to sampling when ewes were assigned to each PRID treatments. The distribution of taxa across treatments within groups is shown in Supplementary Figure S1.

## 3. Results

After assessing pregnancy tests, fertility rate of inseminated ewes reached 55%. In each PRID treatment group, differences in fertility were observed, being 31% for the probiotic group, 54% for the antibiotic group, 60% for the maltodextrin group and 82% for the control group. AI rams showed fertility rates ranging from 50% to 64%.

### 3.1. Composition of the microbiota

**Figure 1:**
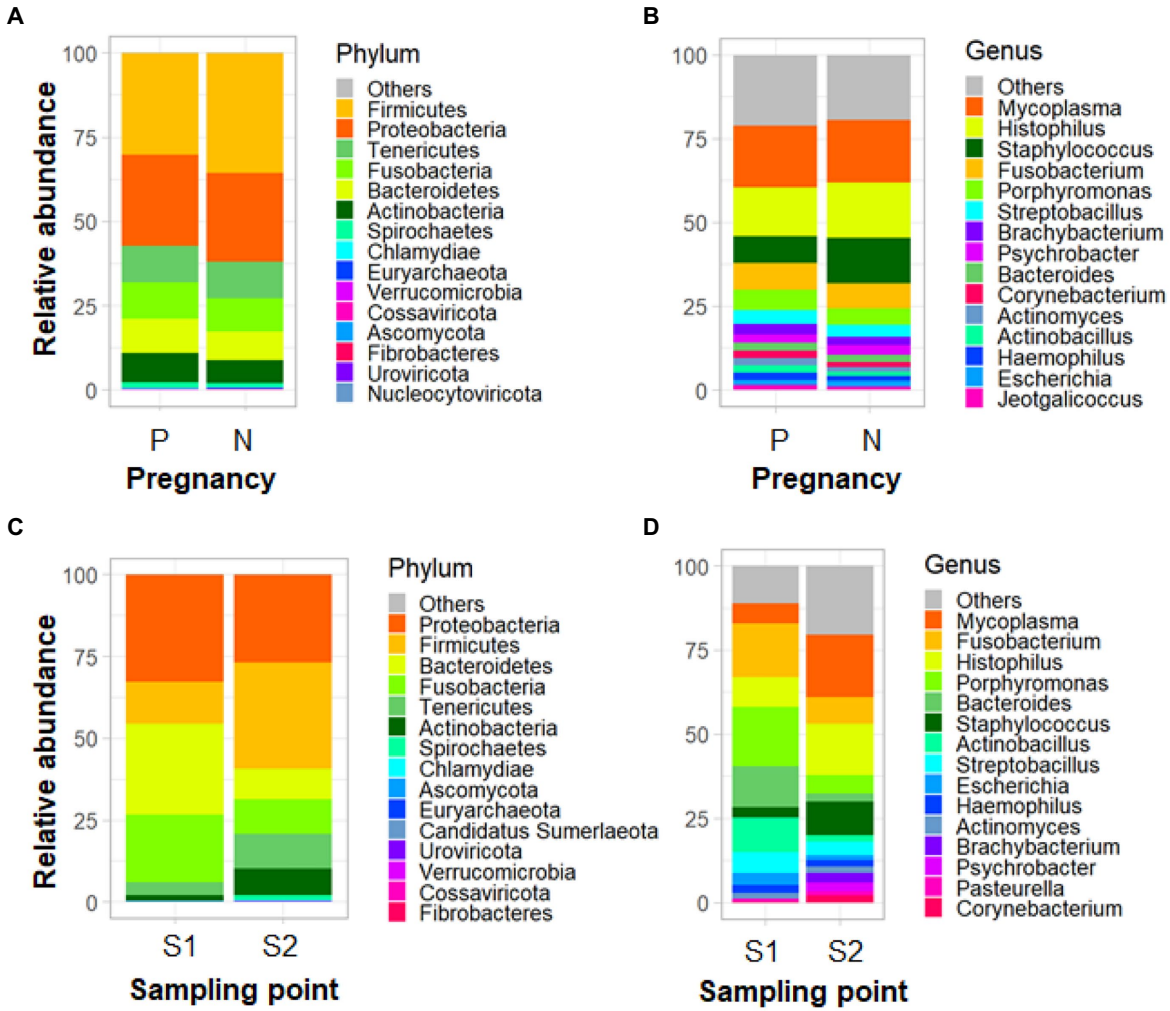
Composition of the microbiota for pregnancy groups (P: positive, N: negative) **(A, B)** and sampling point groups (S1 and S2) **(C, D)** at phylum **(A, C)** and genus **(B, D)** levels.

Before imposing any filtering, the taxonomic composition was as follows: archaea (0.018%), bacteria (20.12%), eukaryota (3.02%), virus (0.03%), and unclassified (76.81%) (most of these unclassified taxa showed some proportion of identity with the host genome, thus suggesting that may be host genome contamination, as expected). In terms of relative abundance, the most abundant phyla detected in the ewes’ vaginal samples were Firmicutes (36.40%), Proteobacteria (26.76%), Fusobacteria (12.05%), Bacteroidetes (9.66%), Tenericutes (7.60%), Actinobacteria (6.01%), Spirochaetes (0.71%), Chlamydiae (0.15%), Ascomycota (Fungi) (0.10%), and Euryarchaeota (Archaea) (0.09%). While the most abundant genera were *Staphylococcus* (17.07%), *Mycoplasma* (10.85%), *Histophilus* (10.22%), *Fusobacterium* (7.37%), *Porphyromonas* (5.51%), *Actinobacillus* (5.12%), *Escherichia* (4.73%), *Streptobacillus* (4.57%), *Bacteroides* (3.97%), and *Ureaplasma* (1.88%). Figure 1 shows microbiota relative abundance at the phylum and genus levels found in pregnant and non-pregnant ewes and in S1 and S2 time points. A similar figure divided by treatment can be found in Supplementary Figure S1.

The five most abundant COGs were ENOG410YQYP (6.77%), ENOG410XST2 (2.94%), ENOG4111K87 (2.92%), COG1132 (1.37%), and ENOG4111K87 (1.16%). The five most abundant KEGGs were K21449 (3.84%), K01154 (1.69%), K0058 (1.31%), K07453 (1.19%), and K07497 (1.05%). Information about the composition of COGs and KEGGs can be found in Supplementary Figure S2.

### 3.2. Alpha-diversity

**Figure 2:**
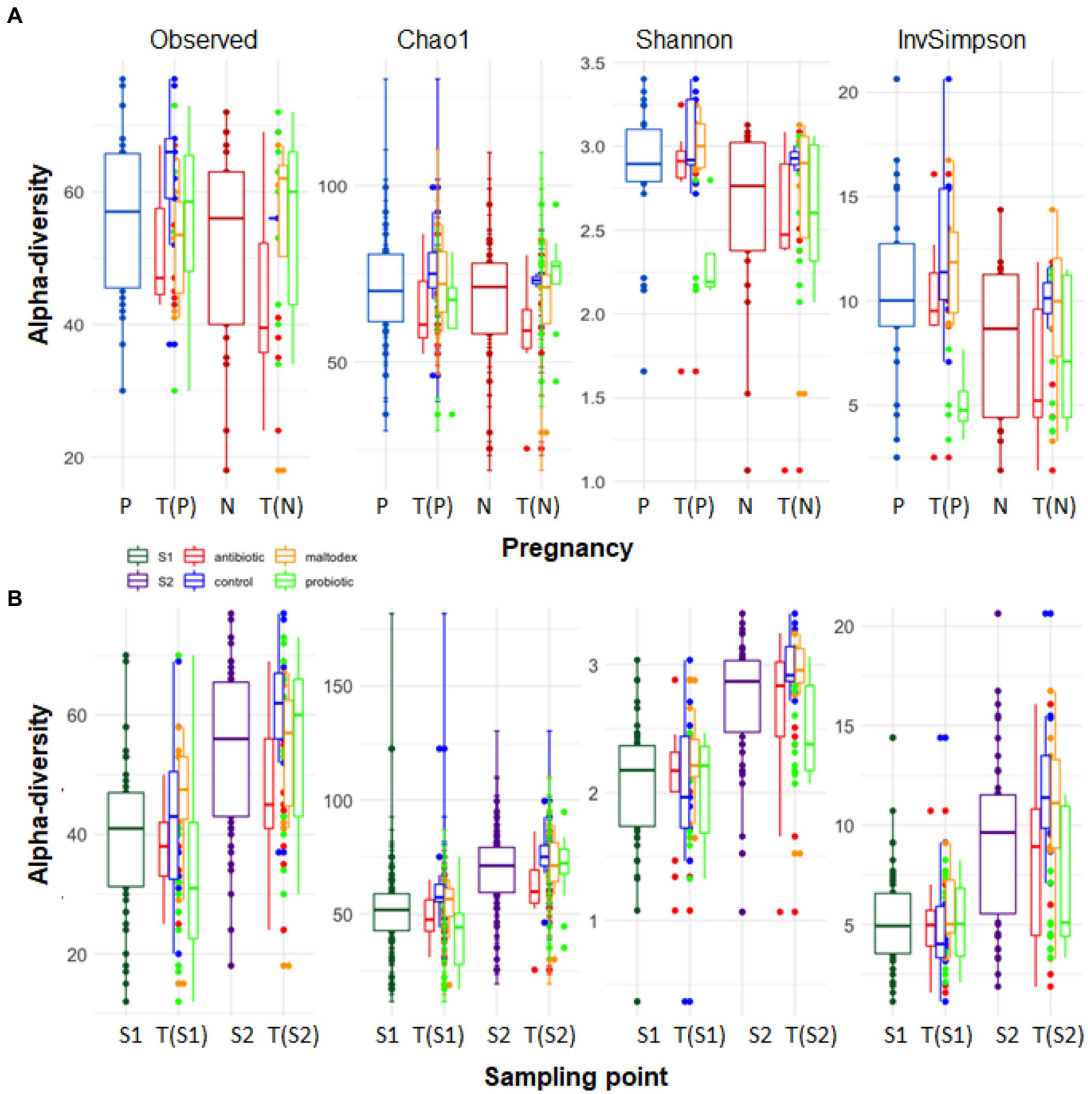
Alpha-diversity measures (observed, Chao1, Shannon, Inverse Simpson index) for pregnancy **(A)** and sampling point groups **(B)** at genus level. P: positive, N: negative, S1: before PRID treatment, S2: after PRID treatment, T(X): treatment within group X.

At the genus level, higher alpha-diversity (although not significant) was observed in pregnant than in non-pregnant ewes for all four measures (Table 1; Figure 2A). Concerning the sampling points, a significantly higher average alpha-diversity, for all metrics studied, was assessed in S2 compared to S1, suggesting an increase of the alpha-diversity as a consequence of the synchronization treatment (Table 1; Figure 2B). Equivalent summaries at the phylum level can be found in Supplementary Figure S3.

**Table 1:** Average alpha-diversity indexes (Observed, Chao1, Shannon and Simpson Inverse) estimated for the microbial composition for pregnancy (P and N) and for sampling time groups S1 (before PRID samples) and S2 (after PRID samples), specifying treatments within each group at the genus level.

	Observed	SD	Chao1	SD	Shannon	SD	InvSimpson	SD
P	56.12	12.34	70.49	14.67	2.84	0.40	10.48	4.27
N	51.62	15.70	66.88	17.45	2.59	0.55	7.92	3.72
								
S1	38.45	14.12	50.15	18.81	2.06	0.52	5.19	2.55
S2	54.11	13.96	68.88	15.90	2.73	0.48	9.34	4.19
								
Antibiotic	48.00	12.92	61.63	15.31	2.61	0.62	8.30	4.22
Control	61.36	11.36	75.58	13.69	3.00	0.23	11.99	3.82
Maltodex	52.40	15.06	69.46	17.00	2.86	0.49	10.93	3.76
Probiotic	55.38	14.49	70.01	16.18	2.53	0.38	6.90	3.25

P: positive for pregnancy, N: negative for pregnancy.

Regarding treatments within S2, the control group presented generalized significantly higher values of alpha-diversity than the antibiotic group. For the other treatments, no significant differences were found.

### 3.3. Beta-diversity

#### 3.3.1. PCA

**Figure 3:**
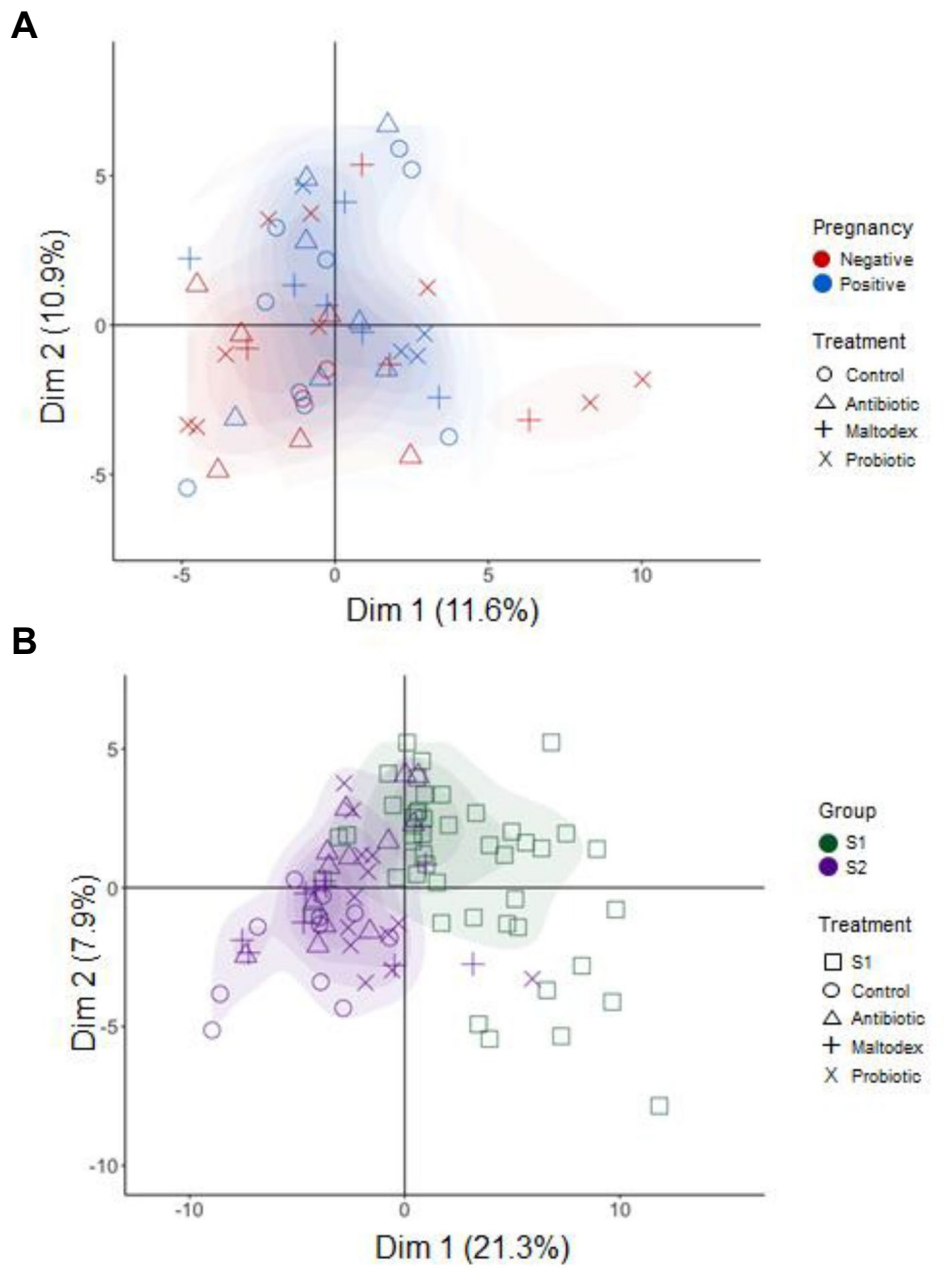
Principal component analysis for pregnancy status **(A)** (negative in red and positive in blue) and sampling time points **(B)** (S1 in green and S2 in purple) groups at genus level. Treatments are represented by different symbols.

At the genus level, no clear differentiation was shown in microbiota diversity between pregnant and non-pregnant ewes (Figure 3A), while there were relevant differences between time points S1 and S2, explaining 21.3% (PCA1) and 7.9% (PCA2) of the total variance (Figure 3B). For COGs and KEGGs no pattern of differentiation was observed between groups.

#### 3.3.2. PERMANOVA

PERMANOVA analysis revealed significant differences in overall microbiota community composition between pregnant and non-pregnant ewes only at the genus level, and no significant differences among PRID treatments at any level. However, highly significant differences were found at the phylum, genus and KEGG categories between sampling points (S1 vs. S2). At the genus and phylum levels, the highest changes observed in beta-diversity were for the control and maltodextrin treatments. At the functional level (KEGG), only for the antibiotic (0.006) and maltodextrin (0.025) treatments, significant changes in beta-diversity between time points S1 and S2 were detected. Table 2 summarizes PERMANOVA results for pregnant vs. non-pregnant ewes and S1 vs. S2 time points, specified by treatment.

**Table 2:** PERMANOVA results for pregnancy (P and N) and sampling time groups (S1 and S2) at genus, phylum and KEGG levels.

		Genus		Phylum		KEGG	
	N (P/N)	MeanSqs	Pr(>F)	MeanSqs	Pr(>F)	MeanSqs	Pr(>F)
P *vs* N	47 (26/21)	160.38	0.021	38.41	0.405	970.16	0.224
Antibiotic	13 (7/6)	112.57	0.408	32.98	0.545	954.91	0.180
Control	11 (9/2)	111.57	0.408	32.91	0.545	954.91	0.180
Maltodextrin	10 (6/4)	99.35	0.541	21.98	0.947	876.98	0.951
Probiotic	13 (4/9)	99.60	0.459	12.44	0.995	756.33	0.857
							
S1 *vs* S2	94	1195.66	0.000	407.08	0.000	1426.78	0.002
Antibiotic	26	372.82	0.000	138.03	0.001	1418.02	0.006
Control	22	487.31	0.000	158.47	0.000	910.68	0.279
Maltodextrin	20	424.39	0.001	144.40	0.000	1046.70	0.025
Probiotic	26	255.02	0.000	73.51	0.044	907.29	0.291

P: positive for pregnancy, N: negative for pregnancy.

### 3.4. Differential abundance analysis

For the analysis of AI success, significant differences between pregnant and non-pregnant ewes were observed only at the genus level. Thus, *Oenococcus* (phylum Firmicutes) and *Neisseria* (phylum Proteobacteria) genera were more abundant in non-pregnant than in pregnant ewes. No significant differences were found among treatments or AI rams. We also explored the *Firmicutes/Bacteroidetes* ratio, related with gut microbial dysbiosis (Grigoreva, 2021), was not significant for any comparison.

**Figure 4:**
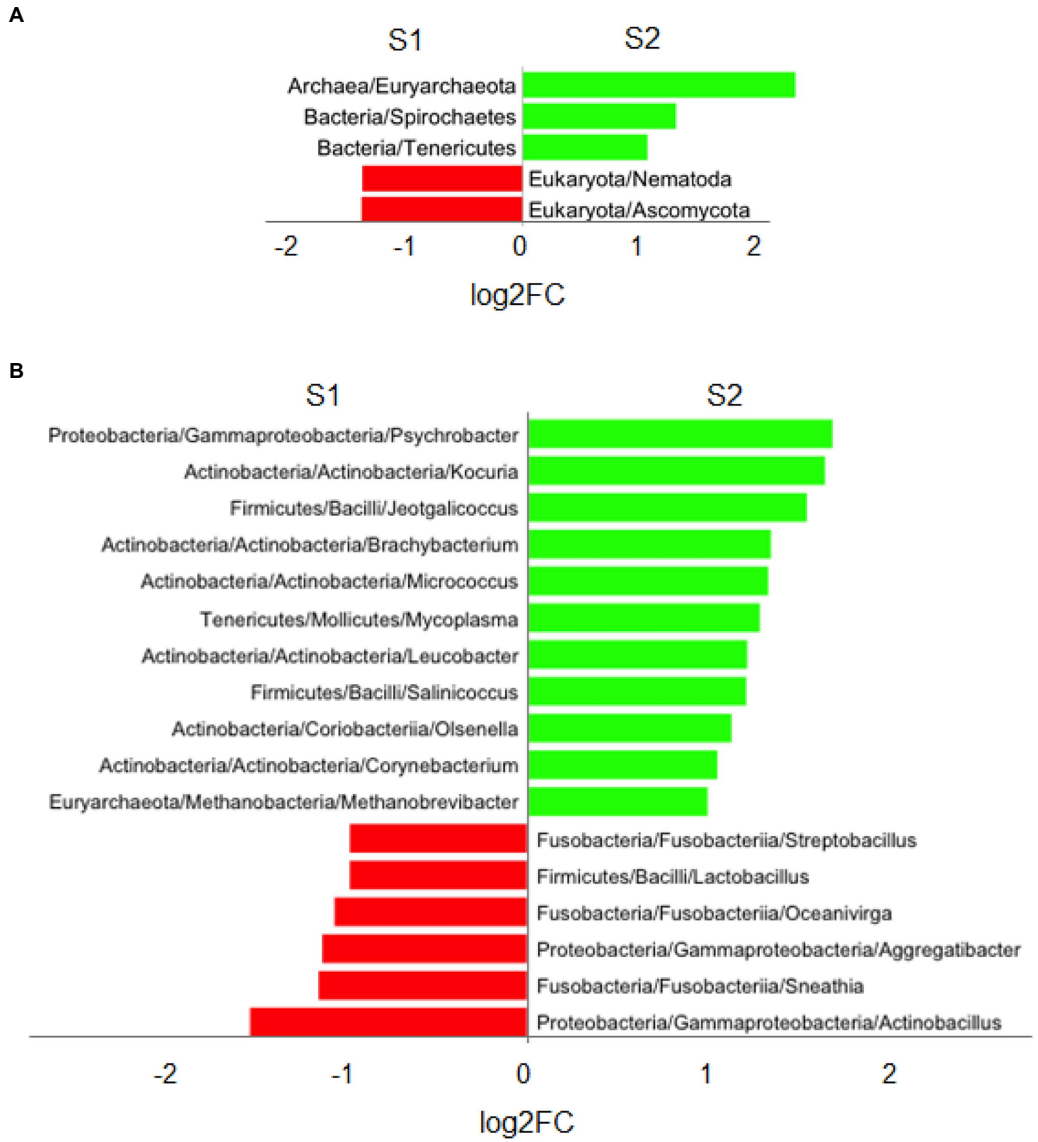
Stacked bar chart showing significant results (FDR at 5%) for differential abundance analysis for sampling point groups S1 and S2 at phylum **(A)** and genus **(B)** levels. A threshold for |log_2_FC| > 1 was imposed for representing the graph. Red represents a higher abundance for S1 compared to S2, while green represents a higher abundance for S2 compared to S1.

For the sampling time comparisons (S1 vs. S2), six and 24 genera were significantly more abundant in S1 compared to S2 group. Among them, the most abundant phyla were Ascomycota and Nematoda. The most abundant genera were *Actinobacillus* and *Aggregatibacter* (phylum Proteobacteria), and *Sneathia* and *Oceanivirga* (phylum Fusobacteria). None COGs or KEGGs showed significant differential abundance between groups. For the S2 group, five phyla (Euryarchaeota, Spirochaetes, Tenericutes, Candidatus Saccharibacteria, Actinobacteria), 25 genera (*Psychrobacter*, *Kocuria*, *Jeotgalicoccus*, *Brachybacterium*, *Micrococcus*, *Mycoplasma*, *Leucobacter*, *Salinicoccus*, *Olsenella*, and *Corynebacterium*, the 10 most abundant), two COG (COG1196 and ENOG410XQ90) and one KEGG (K06919) were significantly more abundant compared to S1. Results from these analyses for phylum and genus are summarized in Figures 4A,B, respectively. Among PRID treatments, many genera in common were observed to decrease and increase between S1 and S2 time points, except for the probiotic-treated samples, where only one genus (*Campylobacter*) decreased and five genera increased from S1 to S2. The antibiotic and maltodextrin treatments promoted the shift of a greater number of genera between S1 and S2, many of them common and others specific to each treatment. The results of the differential abundance analysis within the different treatments is summarized in Supplementary Figure S4A (antibiotic), Supplementary Figure S4B (control), Supplementary Figure S4C (maltodextrin), and Supplementary Figure S4D (probiotic). Supplementary Material also contains detailed results from the differentially abundant analysis between pregnancy groups for phylum (Supplementary Table S1), genus (Supplementary Table S2), COG (Supplementary Table S3) and KEGG (Supplementary Table S4) as well as between timing groups S1 and S2 for phylum (Supplementary Table S5) and genus (Supplementary Table S6).

In order to evaluate whether the samples were a random selection from the population and the potential occurrence of bias in the distribution of samples across groups, comparative analyses were performed within the S1 group (before PRID). Results from these analyses showed non-significant differences between pregnant and non-pregnant groups within S1 (Supplementary Table S7), thus validating the absence of bias due to sampling when ewes were assigned to each PRID treatments. The distribution of taxa across treatments within groups is shown in Supplementary Figure S1.

## 4. Discussion

In the last years, the interest for characterizing vaginal microbiota and its effect on infertility has gained interest. Studies describing microbial populations of livestock reproductive tracts have identified a greater diversity of species compared to humans (e.g., Swartz et al., 2014). In this study, we have investigated the role of vaginal microbiota in sheep AI pregnancy rate and the effect of different treatments added to the progesterone releasing intravaginal devices (PRIDs) for oestrus synchronization in its composition and abundance.

For that, we have used long nanopore technology, which provides a more accurate representation of taxa diversity through the sequencing of whole genomes of a complete microbial community (Kono and Arawaka, 2019; Werner et al., 2022). This third-generation sequencing technique has also the advantage of obtaining information not only from taxonomy but also from gene functions and pathways. To our knowledge, this is the first study addressing the composition and abundance of vaginal microbial communities in livestock using a third generation technology. In addition, one of the interest of our study is that samples were taken in two time points that are crucial: (i) before the administration the PRIDs to the females (this is, the natural microbiota), and (ii) just before AI took place (this is, the modified microbiota as a consequence of the device). In this way, we can investigate not only if the success of AI is affected by microbial composition in the vagina but also the effect of the progestogen (and associated treatments) on the vaginal microbiota. Although recently a body of knowledge has been developed to elucidate the relationship between vaginal microbiome and fertility, all the studies have been carried out through the analysis of the hypervariable regions of the 16S RNA gene (e.g., Swartz et al., 2014 and Serrano et al., 2020 in sheep; Laguardia-Nascimento et al., 2015 in cattle; Mahalingam et al., 2019 in buffalo; Giannatasio-Ferraz et al., 2019 and Quadros et al., 2020 in cows; Rhoades et al., 2021 in macaques).

The current study reveals that the microbial communities identified here are in good agreement with those characterized in previous studies in ovine. So, Firmicutes, Proteobacteria, Fusobacteria, Bacteroidetes, Tenericutes, and Actinobacteria were the predominant phyla in the ewes analyzed. In a previous study of our group, Serrano et al. (2020) found that the highest abundances of ewes vaginal microbiota treated with PRIDs included the phyla Firmicutes, Actinobacteria and Proteobacteria. In the same way, Swartz (2014) found that in sheep vaginal samples, Bacteroidetes, Fusobacteria, and Proteobacteria were the most abundant phyla. At the genus level, the most abundant genera found in our study were *Staphylococcus*, *Mycoplasma*, *Histophilus*, *Fusobacterium*, *Porphyromonas*, *Actinobacillus*, *Escherichia*, *Streptobacillus*, *Bacteroides*, and *Ureaplasma*, also identified in the mentioned studies, although not in the same proportions. Differences among studies can be explained by many factors, some of which are specific to the female such as oestrus cyclicity and pregnancy, but also the type of feeding, the flock management, other environmental factors, the effect of breed itself [see review by Adnane and Chapwanya (2022)], and the own sequencing technique, which is expected to be more accurate since it works with more information than the 16S rRNA gene.

In general, the alpha-diversity was higher in the group of pregnant ewes, for all the alpha-diversity indexes analyzed. Although this difference was not significant, it is in accordance with the results found by Serrano et al. (2020) and Chen et al. (2020) in ewes and dairy cows, respectively, who also observed a higher, but not significant, alpha-diversity associated to reproductive success. Ault et al. (2019) analyzed the vaginal microbiota of beef cows synchronized with a progestogen at different time points at pre-breeding and after pregnancy testing. They observed that non-pregnant cows presented significantly less diversity than pregnant ones at the time of the administration of synchronization devices (day −21), but not in posterior stages. The most recent study analyzing the relationship between vaginal microbiota and pregnancy in sheep was conducted by Koester et al. (2021), who observed a higher microbial diversity in pregnant ewes sampled after lambing, suggesting a more stable environment driven by pregnancy. This finding may however be interpreted with caution, since genital microbiome is diverse among animal species and breeds, and through the different stages of the female reproductive cycle (see Adnane and Chapwanya, 2022). To illustrate, other studies have shown opposite results, with lower alpha diversity in pregnant women (MacIntyre et al., 2015; Freitas et al., 2017; Serrano et al., 2019), or the previously mentioned study in cows by Ault et al. (2019). However, our results are in line with the idea supported by other authors investigating the role of the gut (reviewed by Mosca et al., 2016 and Kriss et al., 2018) and the skin microbiome (reviewed by Carmona-Cruz et al., 2022) in human diseases. This idea states that dysbiosis is linked to a decrease in microbial diversity, due to the increase in pathogenic microbes that compete with other microbial populations, limiting in this way their development. This could be the case of our observation, although it is clear that more research is needed to fully understand the role of microbiome variations (diversity and abundance of specific taxa), as well as the influence of host’s own intrinsic and extrinsic factors, in the pregnancy success.

The analysis of the association between microbial abundance and pregnancy revealed that the genera *Oenococcus* and *Neisseria* were significantly more abundant in non-pregnant ewes compared to pregnant. *Oenococcus* is a lactic acid bacteria responsible for malolactic fermentation. Species from this genus (*O. oeni*) have been isolated in healthy women vaginal samples (Michuki, 2011), where the vaginal tract is typically acidic (pH = 3.5), which is a very different situation from that existing in the vaginal tract of sheep whose pH is neutral. Also in women, a species of *Neisseria* (*N. gonorrhoeae*) has been associated to ectopic pregnancy and infertility (Smolarczyk et al., 2021). In line with this, we also found a lower (although not significant when multitest correction was implemented) abundancy of the genus *Staphylococcus* in the pregnant group. *S. aureus* is a pathogen that causes mastitis and dermatitis in sheep (Yeruham et al., 1999) and a syndrome of lamb pyemia/septicemia (Vautor et al., 2005), and has been reported to cause abortion in sheep (Edwards et al., 2008) and cow (Henker et al., 2020). Thus, species from these genera may independently be associated with a pathogenic effect in sheep.

We also observed that the progestogen included in the synchronization sponges significantly increased the alpha-diversity, regardless of the treatment applied to them. This result was expected, since under higher progesterone concentrations, the populations of microbes in the vagina have been described to be relatively larger (Laguardia-Nascimento et al., 2015). The biological justification for this increase in microorganisms as a result of the progesterone treatment, may be related with the increased levels of glycogen, as suggested by Kaur et al. (2020) in a recent study in humans. The authors observed higher estrogen and progesterone levels at the beginning of the menstrual cycle associated to a higher vaginal bacterial diversity. A similar phenomenon, leading to an increased glycogen production (stimulated through elevated hormone levels), might lead to sudden increase in bacterial diversity. Since glycogen represents a primary nutrient source for vaginal microbes (Mirmonsef et al., 2012), it is likely that increasing progesterone levels in the sinchronizations devices contribute to increase the microbiota diversity. Linking with the aforementioned relationship of the diversity of the vaginal microbiome with pregnancy, it may therefore suggest a beneficial effect of the progesterone included in the PRIDs on the pregnancy outcome, regardless of the treatment applied to them. However, Martinez-Ros et al. (2018) found that the effect of progesterone is changing depending on the time the ewes are exposed to the treatment. The two batches with the highest increase in alpha diversity comparing S1 vs. S2 samples (Table 1) were the control and maltodextrin groups, which were also those showing the better reproductive performances, 82 and 60% pregnancy rates, respectively. This further supports the positive association between a more diverse vaginal microbiome and a successful pregnancy.

In general terms, a significant decrease of microorganisms from the Gammaproteobacteria and Fusobacteria classes and a significant increase of those from Actinobacteria and Firmicutes have been identified in the after PRID group (S2) compared to the before PRID group (S1). Gammaproteobacteria and Fusobacteria include species which have been previously related to fertility failure in ewes (Serrano et al., 2020), such as *Actinobacillus seminis* (Gammaproteobacteria), *Sneathia vaginalis* and *Streptobacillus notomytis* (Fusobacteria), and in women such as *Pasteurella multocida* (Gammaproteobacteria) and *Leptotrichia amnioni* (Fusobacteria) (Waldor et al., 1992 and Gundi et al., 2004, respectively). This finding may suggest that the use of progestogen synchronization devices could be beneficial for improving AI efficiency by decreasing the relative abundance of some pathogenic classes and the increase of favorable taxa. However, a direct association between PRIDs and pregnancy has not been found in our study. In this line, a significant increase in Proteobacteria and Actinobacteria was observed during pregnancy in mice (Koren et al., 2012). Indeed, previous studies have described a probiotic effect for some genera of the Actinobacteria class. These include economically and biotechnologically valuable prokaryotes responsible for the production of bioactive secondary metabolites, notably antibiotics, both antitumor agents and immunosuppressive agents, and enzymes. *Dietzia* genus (family Dietziaceae, order Mycobacteriales, class Actinobacteria) has been reported as probiotic treatment against Jhone disease (paratuberculosis) in cattle (Click and van Kampen, 2009; Click, 2011a, 2011b). This genus appears in significantly higher abundance in the group of ewes whose PRIDs were treated with antibiotic, and which showed a fertility of 54%. In the ewes’ batch treated with maltodextrin, the *Corynebacterium* genus showed a significant overabundance in the S2 samples. The parenteral application of *Corynebacterium cutis* (family Corynebacterineae, order Actinomycetales, class Actinobacteria) in pregnant ewes increased the IgG levels of the dams and their lambs, with positive effects on birth and bodyweight of lambs, decreasing the fetal death rate (Yilmaz et al., 2011). In cows, the Actinobacteria *Kokuria kristinae* (family Micrococcaceae, suborder Micrococcineae, order Actinomycetales, class Actinobacteria) has been shown strong adherence to the vaginal mucus, producing organic acids which can play a role in prevention of unsuitable contamination. This species also presented antimicrobial activity against strains of *Arcanobacterium pyogenes*, *Fusobacterium necrophorum*, *Streptococcus equi* subsp*. zooepidemicus* and *Gardnerella vaginalis* (Styková et al., 2016). In this work, *Kokuria* genus showed a high increase in the after PRIDs samples (S2) subject to antibiotic, maltodextrin and control treatments, but not in the probiotic batch. This fact, together with the lower fertility found in this last group (31%), could lead to hypothesize about a favorable effect of this genus on the ewes pregnancy rate, and perhaps, an antagonistic effect of the probiotic (*Lactobacillus*) on its abundance. On the other hand, the probiotic group was the only one that showed a significant increase of the *Mycoplasma* genus (log_2_FC = 2.2) in the samples collected after the withdrawal of the PRIDs (S2). This genus includes the species *Mycoplasma genitalium,* a sexually transmitted pathogen, which has been identified in women in association with *Chlamydia trachomatis* and *Neisseria gonorrhoeae*, both taxa related to infertility (Sameni et al., 2022).

Regarding the probiotic treatment, the reason to use a *Lactobacillus* species was based on its capacity to initially lower the vaginal pH to avoid the proliferation of pathogenic bacteria. In this study, the species used as probiotic treatment was *Lactobacillus rhamnosus*, since previous studies in humans (Reid et al., 1995, 2001; Cribby et al., 2008; Kirjavainen et al., 2008; Bertuccini et al., 2017; Ratner et al., 2019) and other livestock species (Ametaj et al., 2014; Swartz et al., 2014; Breshears et al., 2015; Deng et al., 2015; Colombo et al., 2018; Niu et al., 2019) have shown its positive effect on fertility. In a recent study by Quereda et al. (2020) in ewes, a vaginal infusion designed with a combination of probiotic *Lactobacillus* species (60% *Lactobacillus crispatus*, 20% *Lactobacillus brevis* and 20% *Lactobacillus gasseri*) at the time of the insertion of fluorogestone acetate sponges for oestrus synchronization, seemed to show a tendency toward the improvement of fertility (60% vs. 91%). In this work, the apparent lack of probiotic effect on fertility, could be the consequence of low colonization rates of probiotic strains isolated from niches other than the ewe reproductive tract (Chenoll et al., 2019), the suboptimal concentration of the probiotic applied, but also, could be related with the neutral pH (7.5) of the sheep vaginal tract that could prevent the growth of acidophilic bacteria (Swartz et al., 2014). Ametaj et al. (2014) proved the efficiency of a combination of two lactic acid bacteria species, *Lactobacillus sakei* and *Pediococcus acidilactici*, isolated from the vaginal tract of healthy cows to decrease the incidence of metritis, pyometra and vaginal purulent discharges. In cows, a combination of *Lactobacillus rhamnosus*, *Pediococcus acidilactici* and *Lactobacillus reuteri* reduced the *Escherichia coli* infection in uterus and the acute inflammation associated (Genis et al., 2017). On the other hand, optimal pH for sperm viability ranges from 7.0 to 8.5, and a reduction in sperm motility occurs at pH of less than 6.0. In this sense, a reduction in the vaginal pH (pH was not measured) as a consequence of the probiotic treatment, could have a negative effect on sperm viability and therefore on the reproductive success. However, a recent study showed that the presence of *Lactobacillus* species at a concentration of 1 × 10^8 CFU had the potential to restrain lipid peroxidation and significantly maintain sperm motility and viability under induced oxidative stress (Tvrdá et al., 2022). Notwithstanding, a study by Saleh et al. (2014) in sheep, found that the presence of *Campylobacter* in vaginal microbiota was associated with increased rates of abortion. Here, we found a decrease of this genus in the probiotic treatment, thus suggesting that a beneficial effect was produced (Hashem and Gonzalez-Bulnes, 2022).

The antibiotic group presented generalized significantly lower values of alpha-diversity than the control group. This result was expected and is in line with previous literature. For example, in cattle with metritis treated with antibiotics, the diversity of the microbiome was decreased after treatment independent of treatment type and cure status (Zhou et al., 2017). Several studies have confirmed the beneficial effects of antibiotics to address several reproductive microbial infectious diseases and related infertility problems (Cicinelli et al., 2018; Zhang et al., 2019). Despite these benefits, treatment with antibiotics can negatively affect fertility of males and females, specially affecting sperm cells and the male reproductive tract (Silla et al., 2015). Additionally, the abusive use of antibiotics poses a serious health risk due to the development of multiple-antibiotic-resistant microbial species. The use of antibiotics in the progestogen releasing intravaginal devices is common in livestock reproductive management. In this study, the ewe’s batch carrying PRID traded with antibiotic showed a pregnancy rate of 54%, the second lowest of the whole experiment. Despite a decrease of potential pathogens related with fertility such as *Actinobacillus*, *Streptobacillus*, *Pasteurella*, *Haemophilus*, *Sneathia*, *Aggregatibacter* and *Mannheimi*a genera was observed between S1 and S2 time points in the antibiotic group, no beneficial effect on fertility appears to have been produced by the use of the antibiotic.

The analysis of genes and pathways (COGs and KEGGs) revealed a significantly higher abundance of the K06919 pathway (putative DNA primase/helicase) in the after PRID samples group (S2), which is involved in prokaryotic defense against foreign genetic elements. Since it is an essential enzyme for phage DNA replication (Wang et al., 2020), its increase after the removal of the synchronization devices could be responsible for the decrease of harmful classes of bacteria such as Gammaproteobacteria and Fusobacteriia. Interestingly, these bacterial classes have more than 10 genes related to helicase and primase activities. The cluster of orthologous genes COG1196 (chromosome segregating ATPase SMC) was significantly increased in the after PRID samples (S2), and it is involved in chromosome condensation, cohesion and repair. In line with this, the superfamily of ATPases include ABC transporters and the repair protein Rad50 (Hirano, 2005), which have been associated to essential structural functions in bacteria (Graumann, 2001). In addition, Actinobacteria (Actinobacteria) and Bacilli (Firmicutes) classes contain 270 and 190 genes, respectively, belonging to this category of orthologous genes. Finally, a Clathrin assembly protein (ENOG410XQ90) involved in intracellular trafficking, secretion and vesicular transport but also in defense mechanisms (Liu et al., 2021) also was found increased in the after PRID samples group (S2), suggesting that also at the level of genes and pathways the effect of PRID seems to be beneficial.

In conclusion, the use of long-reading nanopore technology, has allowed us to confirm the great microbiological diversity existing in the reproductive tract of sheep. We have found a higher relative abundance of the genera *Neisseri*a (Proteobacteria) and *Oenococcus* (Firmicutes) in non-pregnant ewes that may contain potentially pathogenic species related to reproductive failure. We also observed an increase of the microbial diversity and a general decline of the classes Gammaproteobacteria and Fusobacteriia and increase of the classes Actinobacteria and Bacilli in the samples collected after oestrus synchronization devices (PRIDs) removal, which suggest that the microbiological conditions at the time of insemination may exert a certain influence on reproductive success/failure. In general, although our results are not concluding, they support the idea of a beneficial effect of PRIDs on the composition of the vaginal microbiota irrespective of the treatment used (in no case a detrimental effect). This is relevant, since the use of heat synchronization devices in artificial insemination of dairy sheep is an essential strategy, not only to concentrate lambing over time, which ensures large batches of lactating females, but also, because insemination is performed *via* cervical with fresh semen, which requires large batches of females in heat. Finally, the species related to improved pregnancy rate identified in this study could be used as candidates to design specific probiotics for further research.

## Data availability statement

The data presented in the study are deposited in the NCBI-SRA (Sequence Read Archive) repository, accession number PRJNA939000.

## Ethics statement

The animal study was reviewed and approved by The current study was carried out under a Project License from the INIA Scientific Ethic Committee. Animal manipulations were performed according to the Spanish Policy for Animal Protection RD 53/2013, which meets the European Union Directive 86/609 about the protection of animals used in experimentation. We hereby confirm that the INIA Scientific Ethic Committee (IACUC) has approved this study. Written informed consent was obtained from the owners for the participation of their animals in this study.

## Author contributions

MSe, JC, and Msa contributed to the conception and design of the study. CG assisted with laboratory work and organized the database. ER-P, MSa, OG-R, AF, RP-P, AL-G, AS-M, and MSe performed the statistical analysis. ER-P, MSa, and MSe wrote the first draft of the manuscript. All authors contributed to the article and approved the submitted version.

## Funding

This work was funded by INIA-GENOVIS (grant CON19-043-MGA), Ministerio de Economía y Competitividad, Spain (grant RTI-2018-096487-R-C33), and also has received financial support from Fondos FEDER.

## Conflict of interest

The authors declare that the research was conducted in the absence of any commercial or financial relationships that could be construed as a potential conflict of interest.

## Publisher’s note

All claims expressed in this article are solely those of the authors and do not necessarily represent those of their affiliated organizations, or those of the publisher, the editors and the reviewers. Any product that may be evaluated in this article, or claim that may be made by its manufacturer, is not guaranteed or endorsed by the publisher.

## Footnotes

^1^ https://bio.tools/bbmap
