# Supplementary material for: Impact of oestrus synchronization devices on ewes vaginal microbiota and artificial insemination outcome
Source: Front Microbiol. 2023 Mar 23;14:1063807. doi: 10.3389/fmicb.2023.1063807 (PMC10076614; doi:10.3389/fmicb.2023.1063807)
Supplement: Supplementary file 8 [file Data_Sheet_1.PDF]

Supplementary Figure S1. Composition of the microbiota for pregnancy groups (Positive, Negative) (A, B), and sampling point groups (S1 and S2) (C, D) at phylum (A, C) and genus (B, D) levels, differentiating by treatment applied to the PRID (A: antibiotic, C: control, M: maltodextrin, P: probiotic). S1: before PRID administration, S2: after PRID removal.

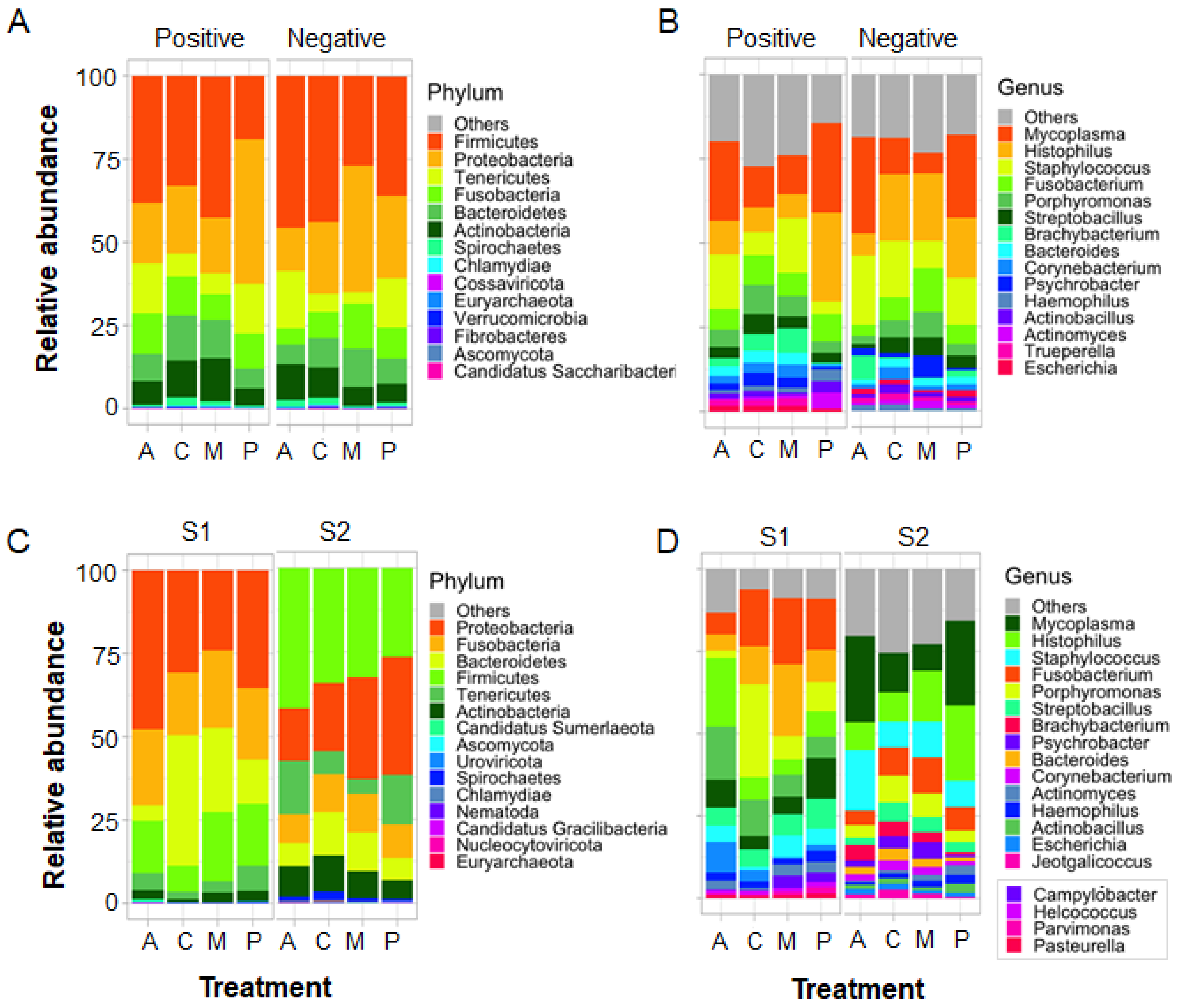

Supplementary Figure S2. Composition of the most abundant (relative abundance) COG (A, C) and KEGG (B, D) for pregnancy groups (P: positive, N: negative) (A, B) and sampling point groups (S1 and S2) (C, D). S1: before PRID administration, S2: after PRID removal.

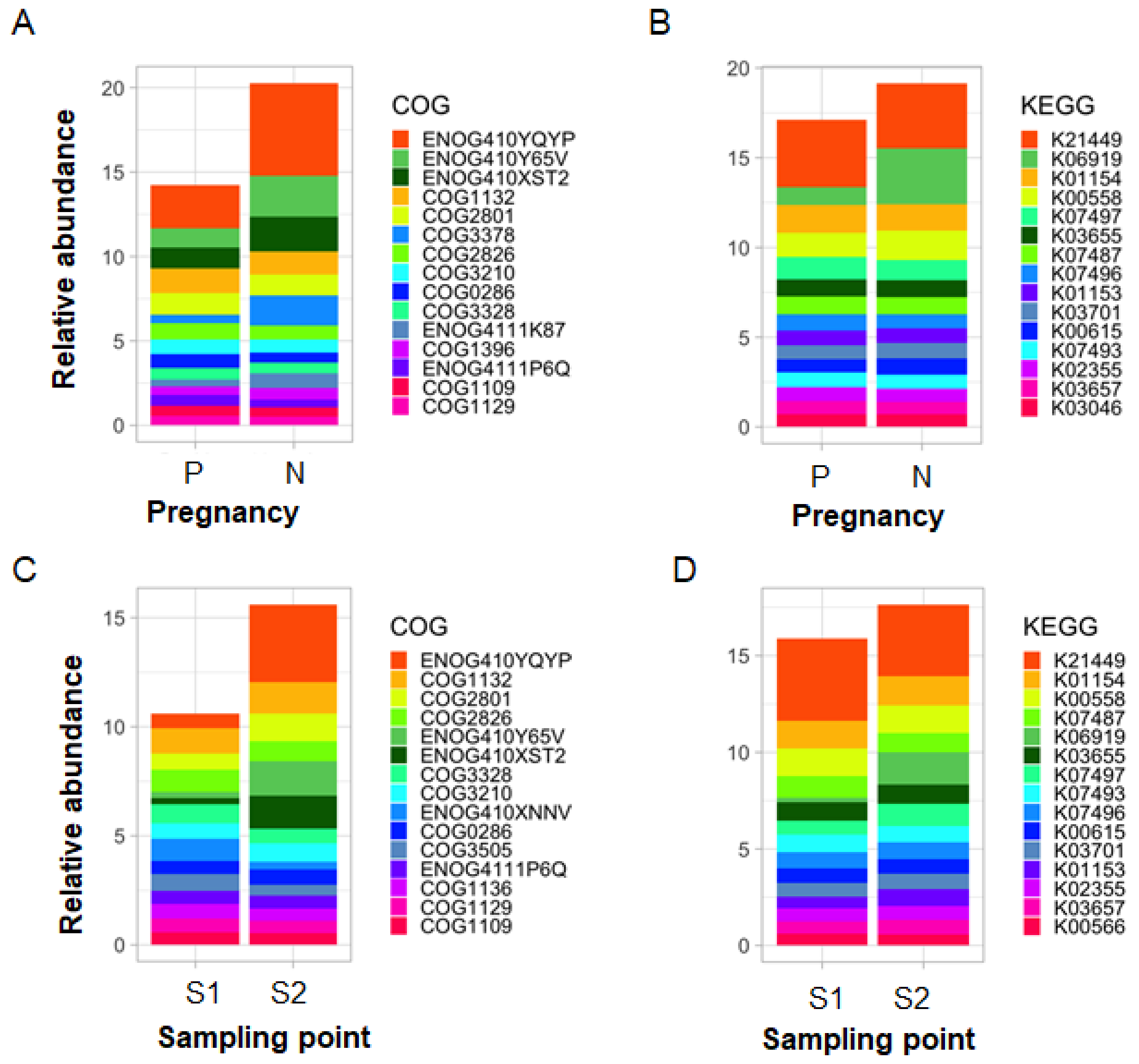

The five most abundant COGs were ENOG410YQYP (name and function unknown), ENOG410Y65V (retrotransposon protein), ENOG410XST2 (cell surface proteoglycan that bears heparan sulfate), ENOG4111K87 (name and function unknown), and COG1132 (ABC-type multidrug transport system, ATPase and permease components).

The five most abundant KEGGs were K21449 (trimetric autotransporter adhesin), K01154 (type I restriction enzyme, S subunit), K0058 (D-3-phosphoglycerate dehydrogenase/2-oxoglutarate reductase), K07453 (putative restriction endonuclease), and K07497 (putative transposase).

Supplementary Figure S3. Alpha-diversity measures (observed, Chao1, Shannon, Inverse Simpson index) for pregnancy (A) and sampling point groups (B) at phylum level. P: positive, N: negative, S1: before PRID administration, S2: after PRID removal, T(X): treatment within group X.

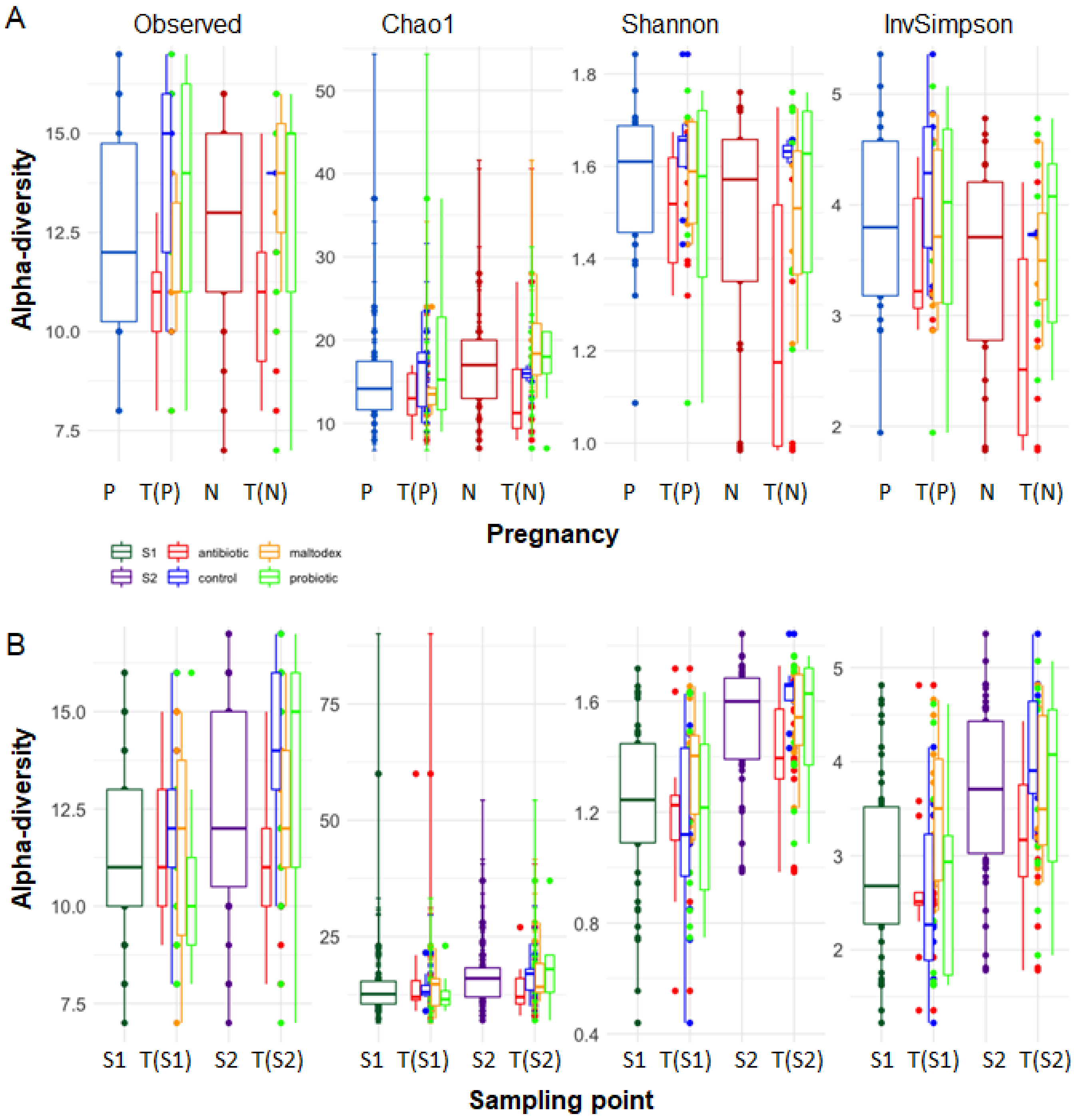

Supplementary Figure S4. Stacked bar charts showing significant results (FDR at 5%) for differential abundance analysis for sampling point groups S1 and S2 at genus level for the antibiotic (A), control (B), maltodextrin (C) and probiotic (D) groups. S1: before PRID administration, S2: after PRID removal.

A

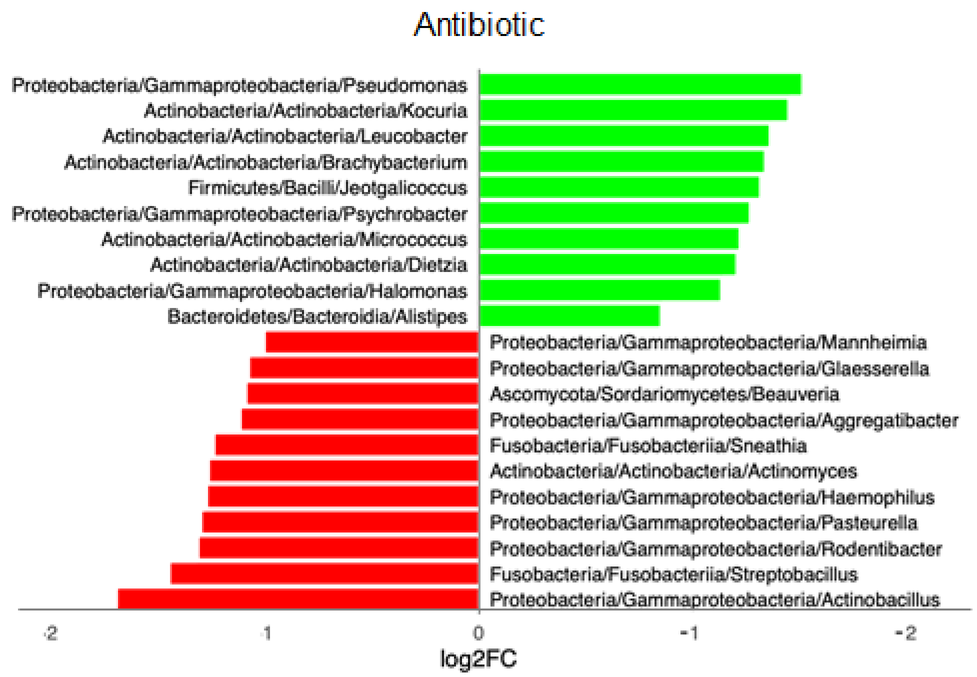

Considering treatments, samples treated with **antibiotic** showed three phyla (Ascomycota, Nematoda, Proteobacteria) and 11 genera (*Actinobacillus*, *Streptobacillus*, *Rodentibacter*, *Pasteurella*, *Haemophilus*, *Actinomyces*, *Sneathia*, *Aggregatibacter*, *Beauveria*, *Glaesserella, and Mannheimia*) significantly more abundant in S1 compared to S2 group. For S2, one phylum (Fibrobacteres), 10 genera (*Pseudomonas, Kocuria, Leucobacter, Brachybacterium, Jeotgalicoccus, Psychrobacter, Micrococcus, Dietzia, Halomonas, and Alistipes*), and one KEGG (K18118 f) were significantly more abundant compared to S1.

B

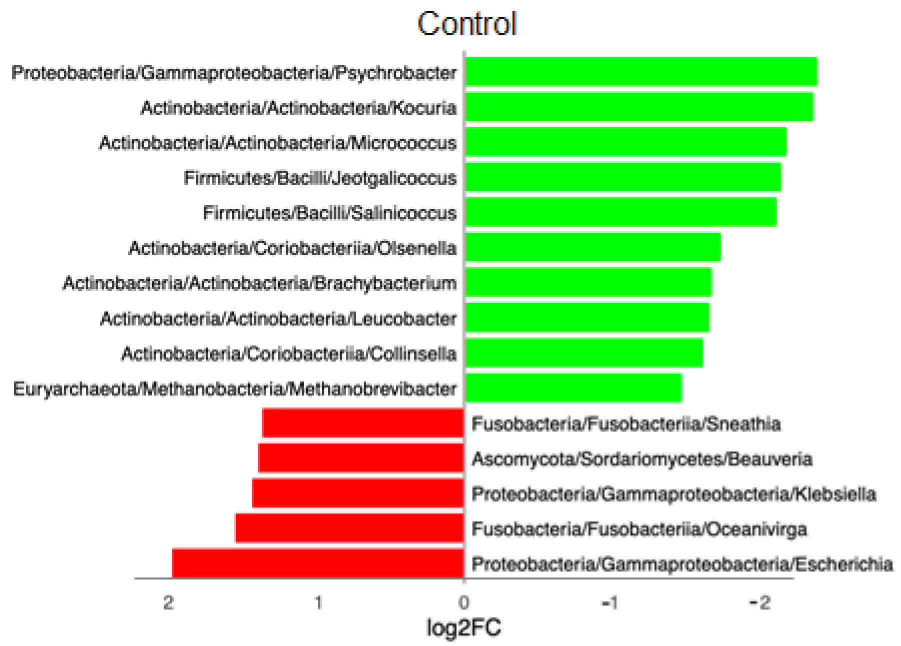

For the **control** samples, three phyla (Ascomycota, Nematoda, Candidatus Peregrinibacteria) and five genera (*Escherichia*, *Oceanivirga*, *Klebsiella*, *Beauveria*, *Sneathia*) were significantly more abundant in S1 compared to S2. For S2, two phyla (Euryarchaeota and Spirochaetes), 10 genera (*Psychrobacter*, *Kocuria*, *Micrococcus*, *Jeotgalicoccus*, *Salinicoccus*, *Olsenella*, *Brachybacterium*, *Leucobacter*, *Collinsella*, *Methanobrevibacter*), and one KEGG were significantly more abundant than in S1.

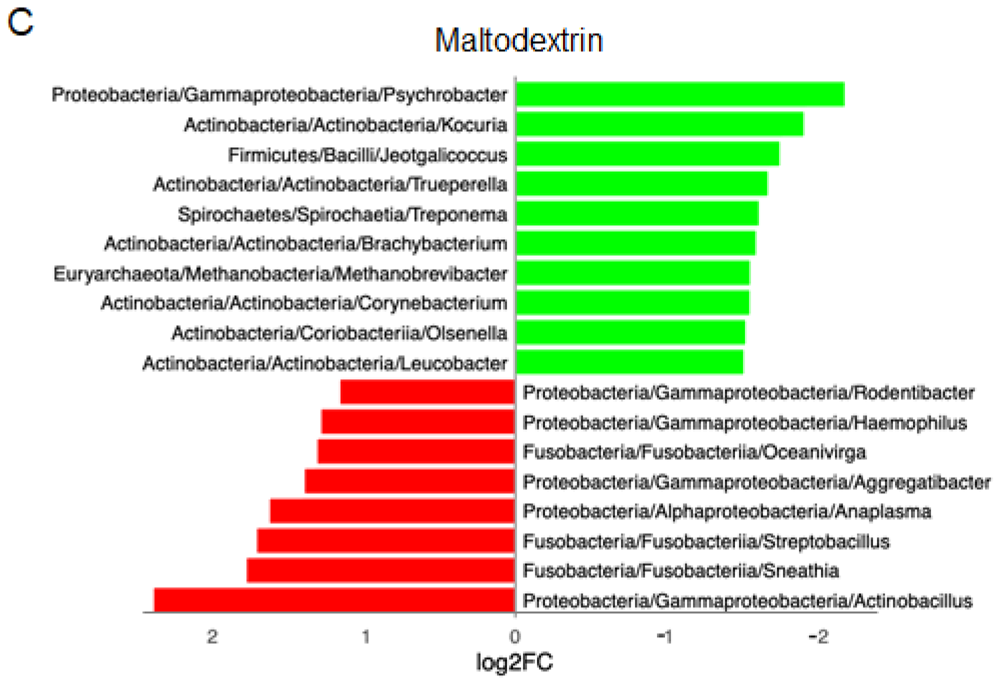

For the **maltodextrin** treatment, two phyla (Ascomycota and Proteobacteria phylum), eight genera (*Actinobacillus*, *Sneathia*, *Streptobacillus*, *Anaplasma*, *Aggregatibacter*, *Oceanivirga*, *Haemophilus*, and *Rodentibacter*), and one KEGG (K09963) were significantly more abundant in S1 compared to S2. For S2, and one phylum (Euryarchaeota) and 10 genera (*Psychrobacter*, *Kocuria*, *Jeotgalicoccus*, *Trueperella*, *Treponema*, *Brachybacterium*, *Methanobrevibacter*, *Corynebacterium*, *Olsenella*, *Leucobacter*) were more abundant compared to S1.

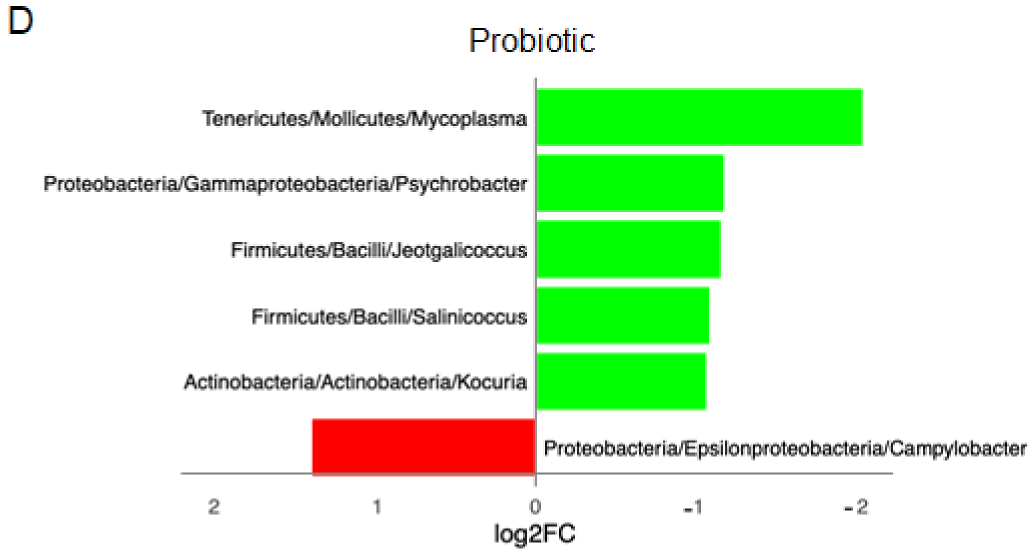

Finally, for the **probiotic** treatment, *Campilobacter* was the only differentially more abundant genus in S1 compared to S2, while for S2, the genera *Mycoplasma*, *Psychrobacter*, *Jeotgallicoccus*, *Salinicoccus*, and *Kocuria* were significantly more abundant compared to S1.
